# Methods for controlling heavy metals in environmental soils based on artificial neural networks

**DOI:** 10.1038/s41598-024-52869-9

**Published:** 2024-01-31

**Authors:** Ninglin Luo

**Affiliations:** College of Architecture Engineering, Chongqing Industry Polytechnic College, Chongqin, 401120 China

**Keywords:** Computational biology and bioinformatics, Environmental sciences, Chemistry

## Abstract

The problem of heavy metal pollution in soil has become a global environmental problem, and it is very important to predict and manage the heavy metals in the environmental soil in a timely manner. The changes in heavy metal content in soil have characteristics such as nonlinearity and large delay, making it difficult to predict heavy metals in soil using traditional methods. Traditional prediction methods are complex and cumbersome, which can lead to longer treatment time and easy secondary pollution. This article analyzed the Back Propagation neural network (BPNN) in artificial neural networks (ANN) and applied it to the prediction of heavy metals in environmental soils. BPNN has good nonlinear function approximation ability, so it can be well applied to complex problems such as soil heavy metal prediction. The methods of treating soil heavy metals include physical repair method, chemical repair method, microbial repair method, plant repair method, plant microbial combined repair method and so on. The use of BPNN can predict heavy metals in environmental soils through adaptive dynamic learning. However, the training time of the BPNN is relatively long and the convergence speed is relatively slow. Therefore, additional momentum terms were added to adjust the weights and thresholds of the network to improve the BPNN. In the experiment, the prediction performance of the improved BPNN was compared before and after the improvement. This article took 50 monitoring data of heavy metals in the same soil in a certain region in 2021 as sample data and predicted the content of heavy metals in the soil using improved and improved BPNN. Due to time constraints, this article only conducted experimental analysis on heavy metals such as lead and cadmium. In the first experiment, when the soil sample data was 50, the prediction accuracy of the BPNN for cadmium before and after improvement was 75.95% and 89.56%, respectively. In the second experiment, when the soil sample data was 50, the prediction accuracy of the BPNN for cadmium before and after improvement was 77.99% and 89.85%, respectively. The improved BPNN has good prediction accuracy and can effectively predict the status of heavy metals in soil. The analysis in this article can provide scientific basis for the comprehensive prevention and control of heavy metals in regional soil, and also provide reference for the development of pollution-free agriculture and ensuring food safety.

## Introduction

As China’s social and economic development continues, some chemical companies have greater economic benefits. While increasing production and processing efforts, they also discharge a large amount of heavy metal pollutants into the soil. However, soil is the material basis for human survival, so heavy metal pollution in soil poses a huge threat to human life. Traditionally, people first detect heavy metals in soil and then treat them based on actual conditions. This method is costly and has poor treatment effects. Moreover, testing involves collecting samples from the site and measuring them through optical, biological, and electrochemical methods, but there are issues such as cumbersome and time-consuming pre-treatment steps. A Backpropagation Neural Network (BPNN) is an artificial neural network commonly used to solve various pattern recognition and prediction problems. In the treatment of heavy metals in environmental soil, BPNN method can be used in the following aspects.

- Prediction of soil heavy metals: BPNN can be used to predict soil heavy metals content. By inputting the physical and chemical properties of a soil sample, as well as environmental factors, the BPNN can train a model to predict the amount of different heavy metals in the soil. This facilitates early detection of areas where excessive heavy metals are present so that control measures can be taken.
- Soil heavy metal monitoring: BPNN can be used for real-time soil heavy metal monitoring. A network of sensors can collect data on soil samples, which are then fed into BPNN for analysis. If excessive heavy metals are detected, the system can immediately issue an alarm, which helps to take timely control measures.
- Soil heavy metal remediation planning: BPNN can be used to plan soil heavy metal remediation strategies. By inputting different remediation methods and conditions, BPNN can predict which remediation strategy will work best for a particular soil sample. This helps to rationally plan and optimize the restoration process to reduce costs and increase efficiency.

Therefore, conventional testing methods are not feasible for controlling heavy metals in actual environmental soils. Jiang Ling found that agricultural land in many regions of China has been contaminated by heavy metals caused by mining activities. He investigated an in situ remediation program applicable to the restoration of heavy metal contaminated agricultural soils around mining sites and conducted a 1-year field trial at a gold mining site to evaluate the effectiveness of soil restoration measures to ameliorate heavy metal contaminants in surrounding contaminated agricultural soils^1^. Shafiq Mohammad studied the absorption, growth, and protein expression of heavy metals in two tobacco varieties. His statistical analysis of data showed that the growth parameters of tobacco were related to the accumulation of heavy metals^2^. Sodango Terefe Hanchiso found that in the past China has undergone a fast industrial revolution and urbanization, and that this expansion has largely led to the release of large amounts of heavy metals into the soil, raising increasing concerns about their potential impact on human health and the environment. However, most of these researches are small in scale, so it is challenging to get a comprehensive picture of the pollution levels in the whole country^3^. The above scholars believe that heavy metal pollution in soil is a persistent, high residue, difficult to degrade, and difficult to repair environment. It can be transmitted to animals through the food chain, posing a serious threat to human health.

As the economy continues to develop and society progresses, pollution problems such as excessive automobile exhaust, factory pollution, untreated discharge of industrial, agricultural, and domestic sewage continue to increase, resulting in increasingly serious soil heavy metal pollution problems. To address these issues, many scholars have proposed the use of artificial neural networks for the prediction of soil heavy metals. Janet Jon Paul used an ANN model to train data, which can solve the problem of quantifying the uncertainty of predicting heavy metals. He overcame these limitations by applying efficient global optimization with a multidimensional expectation improvement criterion^4^. Li Panpan found that the concentration of heavy metals in the soil-rice system was affected by various factors, which hindered the prediction of heavy metal concentrations. He constructed a heavy metal concentration prediction model for rice on the basis of convolutional neural network technique and environmental factors. In addition, he also studied the sensitivity of cellular neural networks to various environmental factors. The findings indicated that the predictive performance of convolutional neural network technology was good^5^. Boudaghpour Siamak found that the supply of safe drinking water is one of the most important issues in society today due to soil heavy metal issues. Through case studies of heavy metals such as lead, zinc, and arsenic, he used neural networks to provide a method for determining the diffusion trend of groundwater pollutants. Only when the extraction rate drops to half can the plain conditions remain stable and the metal concentration does not increase^6^. The above scholars suggest that in order to effectively and economically repair soil, it is necessary to use artificial neural networks to better understand the repair procedures and various options available at different repair stages.

Compared with numerous soil heavy metal prediction models, including some that use statistical methods or linear regression, BPNN based models have unique advantages in several aspects. First, BPNN is a powerful machine learning tool capable of handling highly nonlinear data relationships, which is important in complex soil environments. Secondly, BPNN is adaptive and can continuously optimize the model through training data, thus improving the prediction accuracy. In addition, the capability of BPNN can be further improved by increasing the depth and complexity of the network, making it suitable for a variety of soil environments. Most importantly, the BPNN model has been extensively studied and applied, with rich experience and experimental validation. Therefore, the BPN-based model has shown excellent performance in predicting heavy metal content in soil, providing a powerful tool for solving heavy metal pollution problems.

Heavy metal pollution in soil has problems such as persistence, high residue, high toxicity, difficulty in remediation in a short period of time, and serious impact on agricultural production and food safety. For this reason, there is an urgent need to carry out study on the treatment of heavy metals in soil in order to improve grain quality and reduce economic losses. Artificial neural networks, on the other hand, are a method used for information processing, which mimics some basic structures and features of the human brain to achieve some unique functions of the human brain. For example, the distributed storage and processing of information, as well as intelligent functions such as self-learning and adaptation, have very powerful information processing capabilities, providing a new method for predicting and controlling heavy metal content in soil.

The main contribution of this paper is the application of BPNN to the prediction of heavy metal content in environmental soils. Although BPNN as a neural network model has a certain history in the field of computer science, its application in the field of environmental soil science is relatively new. Through its nonlinear function approximation capability, BPNN provides an effective tool to predict heavy metal content in soil, which is of great significance for soil heavy metal control and environmental protection. In this paper, the application of BPNN is emphasized to solve the complex nonlinear characteristics of heavy metal content in soil. By improving BPNN, the weight and threshold of the network are adjusted by adding additional momentum terms, so as to improve the convergence speed and performance of the model. Although the application of BPNN in soil heavy metal prediction is not new, the method for improving the model to improve training efficiency and prediction performance is an innovation in this paper. Based on the soil monitoring data of a region in 2021, the experiment focused on the prediction of two heavy metals (lead and cadmium). The experimental results show that the improved BPNN shows higher prediction accuracy under different conditions (sample data is 50), which highlights the practical application potential of BPNN in soil heavy metal control, and also emphasizes the practical application of this paper. The research aims to provide scientific basis for the comprehensive control of heavy metals in soil, promote the development of pollution-free agriculture, and ensure food safety.

## Methods for predicting heavy metals in soil based on artificial neural networks

Soil heavy metal pollution is a hidden, harmful, long latency, and irreversible problem. In the food web, heavy metals accumulate in the food web, and finally enter the body, causing serious harm to the human body^7,8^. At the same time, due to heavy metal pollution in the soil, there is less available land, which has had a significant impact on China’s agricultural production. Heavy metals in the soil can also cause pollution of groundwater sources, with serious consequences that directly affect people’s drinking water quality. However, at present, people’s understanding of heavy metals in soil is far from reaching the required level. On the one hand, it is because heavy metals in soil are not easily detected, and on the other hand, it is because people lack sufficient understanding and attention, making the problem of heavy metals in soil increasingly serious. Heavy metals in soil pose a great threat to the sustainable development of society and economy, so effective management and remediation of heavy metals in soil has become an urgent task^9^.

Heavy metals in soil mainly include elements such as mercury, cadmium, lead, chromium, arsenic, copper, and nickel. Heavy metals migrate in the soil and are difficult to biodegrade, and once they enter, they would not disappear^10,11^. Its characteristic is irreversible. Therefore, once the soil is contaminated by heavy metals, it is difficult to treat. Due to the high toxicity of heavy metals, their continuous accumulation in soil can cause soil heavy metals, which in turn pose a threat to human health.

### Heavy metal prediction based on improved BPNN

With the continuous improvement of the living standards of the Chinese people, the content of heavy metals in the soil has attracted more and more attention. Suitable metals can promote the photosynthesis of plants, but excessive metals damage the soil structure, which has a significant inhibitory effect on soil enzyme activity and plant growth. Plants appear browning deformity and uneven growth, causing serious impact on the local ecological environment^12^.

Due to limited time and effort, this article mainly analyzes the hazards of lead and cadmium, two heavy metals. Lead (Pb) is a toxic heavy metal with a bluish gray color. Its main source of pollution is wastewater, waste residue, and exhaust gas discharged by industrial and mining enterprises. Lead and its compounds can enter the body through drinking water and food, and can also enter the lungs through respiration, causing harm to multiple systems such as nerves, hematopoiesis, and kidneys^13,14^. Lead also affects the intellectual development of infants, leading to peripheral neuritis, damage to cerebellum and cerebral cortex cells, and even dementia in serious cases. Cadmium is an important human non-essential metal element that can cause olfactory impairment through airway stimulation, leading to symptoms such as pneumonia and pulmonary edema. Long term consumption of foods containing cadmium and heavy metals can lead to impaired renal tubular function, leading to cartilage damage. Long term cadmium poisoning causes the greatest damage to the kidneys and can also lead to anemia. Therefore, detecting and predicting heavy metals in soil is very essential^15,16^.

Because the prediction of heavy metal content in soil is a multivariate nonlinear problem, using ANN to predict heavy metals in environmental soil is a good solution. BPNN is the most commonly utilized and representative of artificial neural networks. Due to its wide application range, simple operation, and strong scalability, BPNN has been widely utilized in practical applications. This article applies BPNN to the prediction of lead and cadmium in soil^17,18^.

In BPNN (Backpropagation Neural Network), it is crucial to choose the right activation function and transfer function because they play a key role in the model’s performance and training process. Here are some reasons why these functions were chosen as BPNN models.

#### Activation function

##### Nonlinear mapping

The activation function introduces nonlinear mapping to enable neural networks to learn complex nonlinear relationships. Without the activation function, the entire network would perform only linear transformations, limiting its representation capabilities.

#### What the transfer function does

##### Error propagation

The transfer function helps to propagate errors from the output layer back to the network for weight adjustment. It determines how errors propagate backwards in the network, which affects the effectiveness of training.

##### Weight adjustment

The transfer function determines how the weight is adjusted based on the error signal. It can use optimization algorithms such as gradient descent to update the weights of the network, thereby minimizing errors. Each relevant neuron can receive error information and adjust its weight based on the error information. It is in this reciprocating process that the BPNN performs forward operations on information, reversely controls errors, and obtains the expected input results. In the learning process, the BPNN adjusts the weights to get the most precise information by controlling the size of the threshold value^19,20^.

In the forward propagation of information, if $X$, $Y$, and $Z$ are input values, hidden layer calculation results, and output layer calculation results, the formula from input layer to hidden layer is:

1 $$ Y_{b} = f_{1} \left( {\sum\limits_{a = 0}^{l} {V_{ab} \times X_{a} } } \right),\quad b = 1,2, \ldots ,m $$

Among them, $f_{1}$ is the functional relationship, and $V$ is the weight coefficient. The calculation result $Y_{b}$ of the hidden layer is substituted into the calculation formula of the output layer as follows:

2 $$ Z_{c} = f_{2} \left( {\sum\limits_{b = 0}^{m} {W_{bc} \times Y_{b} } } \right),\quad c = 1,2, \ldots ,n $$

Among them, $f_{2}$ is also a functional relationship, which can preliminarily determine the corresponding relationship between input data and calculation results. Due to the frequent errors in forward propagation calculations, the main content of the algorithm feedback mechanism is the correction of errors and the adjustment of weights. If there are a total of $p$ training samples and their expected output value is set to $T_{c}^{p}$, then the error between them and the actual output value $Z_{c}^{p}$ is set to $E_{P}$:

3 $$ E_{P} = \frac{1}{2}\sum\limits_{c = 1}^{n} {\left( {T_{c}^{p} - Z_{c}^{p} } \right)^{2} } $$

The deviation formula for the hidden layer is:

4 $$ \delta_{Rc} = f\left( {Z_{c}^{p} } \right) \times \left( {T_{c}^{p} - Z_{c}^{p} } \right) $$

Error backpropagation is a key step in BPNN, calculating the errors of the network and backpropagating the errors to the layers to update the weights. The loss function is expressed as: $E=L\left(Y,{Y}_{true}\right).$ The chain rule can be used to calculate the error gradient of the output layer and the hidden layer. Output layer error gradient: $\nabla {E}^{(2)}=\frac{\partial E}{\partial {Z}^{(2)}}$, hidden layer error gradient: $\nabla {E}^{(1)}=\frac{\partial E}{\partial {Z}^{(1)}}$, According to the error gradient, the gradient descent optimization algorithm is used to update the weight matrix and the deviation term to reduce the error. The weight update rule is expressed as: output layer weight update: $\nabla {W}^{(2)}=-\alpha \nabla {E}^{(2)}$, hide layer weight updates: $\nabla {W}^{(1)}=-\alpha \nabla {E}^{(1)}$, among them, α is the learning rate and is used to control the step size of weight update. The above forward propagation, error backpropagation and weight updating processes will be iterated repeatedly until the network error reaches a satisfactory degree or a predetermined training round is reached.

The BPNN transforms the data processing process into a class of nonlinear optimal solutions, and on this basis, it is repeatedly trained to reduce errors. According to different data and requirements, the BPNN can set any number of nodes and learning rates in the middle layer, making it more flexible. BPNN can adjust the weight of each neuron through continuous learning, and has strong adaptive ability, good fault tolerance, and a wide range of applications. Nevertheless, the BPNN still has certain shortcomings^21,22^.

The first is that the training takes too long. When dealing with complex problems, the convergence speed of BPNN is slow, and the training speed is affected by the amount of data information, which leads to a long training time for large-scale samples and makes data analysis difficult. The second is the local minimum problem. When the BPNN cannot guarantee its convergence to the optimal solution, local optimization problems occur. BPNN are a class of mathematical optimization problems with strong nonlinearity, which inevitably generate local minima during the solving process. The traditional control method requires an increase in the hierarchy of the BPNN and the number of nodes, but this increases the learning difficulty and time of the BPNN^23,24^.

### Heavy metal prediction based on improved BPNN

The application range of BPNN is the widest, and in the actual use process, many improved algorithms on the basis of BPNN have emerged. Its application in the industry has become increasingly widespread, and the problems solved have become increasingly complex. The biggest problem with traditional BPNN is that the convergence speed of the learning process is relatively slow, and repeated learning is required for the provided learning samples. The number of iterations is usually several hundred to several thousand times to make the error of all output nodes less than the specified allowable error. The improved BPNN adjusts the weights and thresholds of the neural network by introducing momentum terms to accelerate its convergence speed. The reason why models based on BP Neural Network (Backpropagation Neural Network) show better predictive performance in some cases may be due to the following reasons: nonlinear modeling ability: BP neural network is a powerful nonlinear model that can adapt to complex data relationships. This makes it advantageous when dealing with nonlinear problems and data sets with complex patterns. Compared with linear models, BP neural networks can better capture the nonlinear relationship between input features.

Adaptability: BP neural networks can automatically adjust weights and biases through backpropagation algorithms to minimize loss functions. This means that it can adapt to different types of data and problems without having to manually adjust a large number of hyperparameters. The adaptability of BP neural network makes it widely used in various fields.

There are many options for programming languages and software tools for developing BPNN (Backpropagation Neural network) models, using Python for developing and training BPNN models. Python is a very popular programming language with a wide range of machine learning and deep learning libraries such as TensorFlow, Keras, PyTorch, and Scikit-Learn. These libraries provide a wealth of tools and functions for building, training, and evaluating BPNN models.

To enhance the convergence speed of the network, the momentum term is added to adjust the weights and thresholds. The expression formula for the weight adjustment vector containing the momentum term can be written as:

5 $$ \Delta Q\left( t \right) = \eta \delta K + \alpha \Delta Q\left( {t - 1} \right) $$

Therefore, the formula for adjusting the output layer weight by increasing the momentum term is:

6 $$ \Delta q_{jk}^{2} \left( t \right) = \eta \lambda_{k}^{2} + \alpha \Delta Q\left( {t - 1} \right) $$

$\alpha$ is the momentum coefficient, and the weight and threshold adjustment formulas for the output layer and hidden layer of the improved BPNN have been derived. After improving the BPNN, it can be learned that while increasing the momentum term, a portion of the previous weight adjustment is extracted and added to the current weight adjustment.

In practical applications, BPNN with a single hidden layer often have significant network errors and low computational accuracy. People can achieve optimal network error and computational accuracy by increasing the number of hidden layers, which has both advantages and disadvantages. The one-sided increase in the number of hidden layers makes the entire network structure more complex, and the forward propagation and feedback mechanism of the BPNN greatly increases the training time of the network. Usually, Formula (7) is utilized to determine the number of hidden layers.

7 $$ l = \sqrt {m + n} + a $$

If it is only to optimize network error and computational accuracy, it can be achieved by adjusting the number of nodes in the hidden layer. This method is not only simple, but also can better observe and adjust the training effect of the BP (Back Propagation) model. Therefore, without increasing the number of network layers, optimization can usually be achieved by adjusting neural nodes, and it is usually not possible to select too many nodes. Due to the large number of nodes, the network becomes more complex and there are issues such as unclear topology. BPNN is a common type of artificial neural network used to solve a variety of supervised learning tasks. Here are some important details of the BPNN method. Neurons and Hierarchies: BPNN includes three types of neurons: input neurons, hidden neurons, and output neurons. Neurons are organized hierarchically, usually with an input layer, one or more hidden layers, and an output layer.

Weights and biases: there are weights between each neuron to adjust the strength of the signal transmission. Each neuron has a bias (also called a threshold) that controls the output of the activation function.

The activation function is a derivable function that connects the input layer with the hidden layer, and the hidden layer with the output layer. Regardless of whether the BPNN is a hidden layer or multiple hidden layers, they basically choose the sigmoid function as the activation function. This is not only because the sigmoid function is differentiable, but also because it is a continuous differentiable function. The sigmoid function is:

8 $$ f\left( x \right) = \frac{1}{{1 + e^{x} }} $$

The sigmoid function is different from the linear function. The sigmoid activation function needs to be normalized first. The normalized function processing formula is:

9 $$ b = \frac{{a - a_{\min } }}{{a_{\max } - a_{\min } }} $$

In the formula, $b$ represents the normalized data; Input vector $b_{f}$ is input into the BPNN to train and predict the data, and the prediction results are subjected to inverse normalization processing. The formula is:

10 $$ a_{f} = b_{f} \left( {a_{\max } - a_{\min } } \right) $$

The improved BPNN is a typical multi-layer network. Currently, at different levels, neurons at each level appear in a fully connected form, and there is no connection between neurons at the same level. Therefore, the state of neurons at the level only affects neurons at the next level. By utilizing the variation pattern of the weights of each layer in the improved BPNN, the weights of each layer can be adjusted to achieve a high degree of prediction effect. BPNN are a powerful machine learning model, but it also has some shortcomings and limitations, including the following:

- Need a lot of data: BPNN usually needs a lot of data when training to get good performance. For small data sets, BPNN is prone to overfitting, resulting in poor generalization performance.
- Difficulty in selecting hyperparameters: BPNN has multiple hyperparameters, such as the number of layers, the number of neurons, the learning rate, etc. Choosing the right combination of hyperparameters can require a lot of experimentation and adjustment, and there are no universal rules.
- Gradient disappearance and gradient explosion: in deep neural networks, backpropagation algorithms can cause gradient disappearance or gradient explosion problems. This affects the training of the model, especially in deep networks.

## Comparison of the predictive effects of BPNN on heavy metals before and after improvement

### Selection of datasets and parameter settings

#### Dataset

200 sample data from the study area from 2020 to 2022 were selected for analysis. Finally, in order to reduce the differences between experiments, 50 sample data were selected for experiment, and the improved BPNN was utilized to predict them and compare the prediction results. In order to eliminate the large physical quantities and numerical values in the input nodes, the sample data was normalized and improved BPNN were used to predict the effectiveness of soil metals. The article used BPNN before and after improvement to predict the heavy metal content in soil, providing a good basis for governance. The study area is a mining area, and mining activities often lead to soil acidification, as large amounts of sulfuric acid and organic matter enter the soil. Acidic soils help increase the solubility of heavy metals, especially cadmium. The soil in mining areas is often rich in various metallic minerals, including lead, zinc, and cadmium. The presence of these metallic minerals can lead to higher concentrations of heavy metals in the soil. Mining areas usually lack vegetation coverage, which has a certain adsorption and stabilization effect on reducing heavy metal pollution in the soil. The lack of vegetation coverage may lead to greater loss and enrichment of heavy metals in the soil.

When designing a neural network model and dividing the data set into a training set and a test set, the following parameters need to be explicitly specified. Input parameter: this is the input feature or attribute of the model. When dealing with heavy metals in environmental soils, input parameters can include the physical, chemical and environmental properties of the soil, such as pH, organic matter content, particle size distribution, etc. Output parameter: this is the output of the model, that is, the predicted result of the model. When predicting heavy metal content in soil, the output parameter will be the concentration value of various heavy metal elements (such as lead, cadmium, chromium, etc.).

The 200 samples are divided into training sets and test sets. 70% of the samples are used for training and 30% are used for testing. 50 samples were selected in the training set for the experiment. These samples will be used to train and tune the model, which is reserved the remaining samples in the test set. These samples will be used to evaluate the performance and generalization ability of the model. Randomness and uniformity of data should be ensured when dividing data to prevent sampling bias. This can be done through methods such as random sampling or cross-validation.

The performance evaluation of the experiment will use the root mean square error (RMSE) as the evaluation index between the actual value and the predicted value. RMSE is a commonly used regression model performance indicator that measures the standard deviation of a model’s prediction error. A lower RMSE value indicates that the model’s prediction is closer to the actual value, indicating better model performance. RMSE will therefore be used to evaluate the performance of the improved BPNN model and its accuracy in predicting soil heavy metal content. The model errors before and after improvement mentioned in the following paper are calculated as RMSE: $RMSE=\sqrt{\sum {(Y-\widehat{Y})}^{2}/n}$, among them, Y represents the actual value. Ŷ represents the predicted value of the model. Σ represents the sum operation. n is the number of samples.

Determination coefficient is a commonly used regression model evaluation index, which is used in this paper to measure the fitting degree of the model to the actual data, that is, the prediction accuracy of the model in this paper: ${R}^{2}=1-\frac{{RMSE}^{2}}{S}$, among them, S represents the squared difference between the actual value and its mean. The range of values is from 0 to 1. Approaching 1 indicates a good fit to the model, while approaching 0 indicates a poor fit to the model.

#### Parameter settings

Soil environment prediction based on BPNN can be used to estimate the distribution and concentration of heavy metals in soil. This prediction method combines the physical, chemical and environmental properties of the soil to predict the amount of heavy metals in the soil. To compare the predictive effect of BPNN on heavy metals in soil before and after improvement, the selection of the number of hidden layer nodes in BPNN is very important. Therefore, this article conducted a comparative analysis on the selection of hidden layer nodes and obtained parameter settings that meet the prediction requirements. The limit value of prediction error is 1, and the number of hidden layer training times and training time are illustrated in Table 1.

**Table 1:** Hidden layer training error and training time (s).

Hidden layer number	Training frequency	Prediction error	Training time (s)
1	10	0.134	3.65
3	10	0.159	3.78
5	10	0.098	2.55
7	10	0.133	2.91
9	10	0.125	2.57

As shown in Table 1, the number of training sessions for the hidden layer was set to 10. When the hidden layer was set to 1, the prediction error was 0.134, and the training time was 3.65 s. When the hidden layer was set to 9 layers, the prediction error was 0.125 and the training time was 2.57 s. When the hidden layer was set to 5 layers, the prediction error was the smallest at 0.098, and the training time was 2.55 s. The training time was also the shortest.

BPNN can be used to predict the content and distribution of heavy metal elements in soil and to monitor the change trend of heavy metal pollution in soil. Its highly sophisticated pattern recognition and data analysis capabilities enable it to provide more accurate predictions and monitoring results, helping to detect and respond to potential pollution problems early. The determination of hidden layers is a crucial step, as having too few hidden layers can lead to the network falling into local minima. If the number is too large, it increases the training time and difficulty of the network, and there may also be “overfitting” problems. For the hidden layer, to efficiently obtain results while ensuring the accuracy of predicted values, the hidden layer is set to 5 layers.

**Figure 1:**
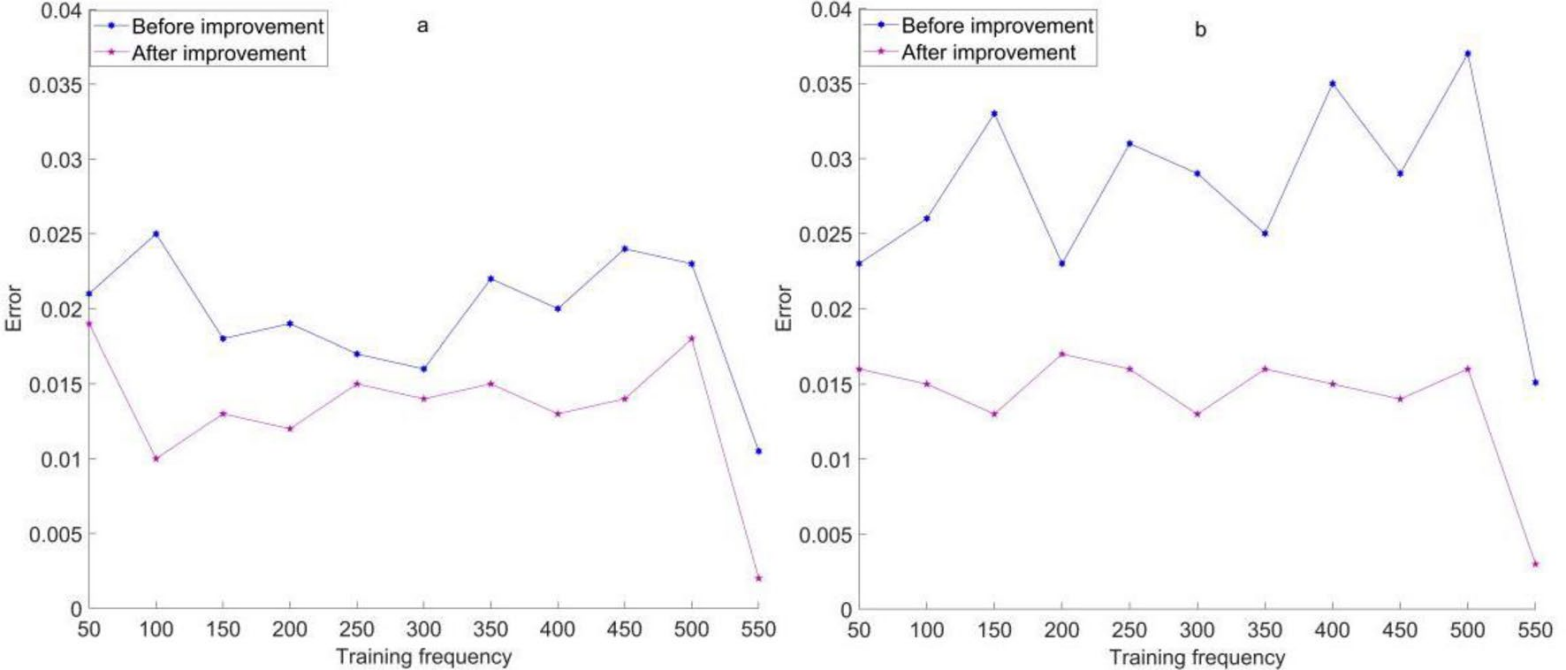
Prediction error of BPNN before and after improvement at 150 and 300 sample points. (**a**) Prediction error of BPNN before and after improvement at 150 sample points. (**b**) Prediction error of BPNN before and after improvement at 300 sample points.

After determining the number of hidden layer neurons, the BPNN before and after improvement was trained, and the prediction of the BPNN before and after improvement was trained. The training frequency was set to 550 times, and 150 sample points and 300 sample points of simulated distribution were selected for analysis. The prediction error of the BPNN before and after improvement under different sample points is illustrated in Fig. 1.

As shown in Fig. 1: in Fig. 1a, when the number of sample points was 150, the prediction errors for the pre improvement and post improvement BPNN training times were 0.021 and 0.019, respectively. When the training frequency of the BPNN before and after improvement was 550, the prediction errors were 0.011 and 0.002, respectively.

In Fig. 1b, it can be seen that when the number of sample points was 300, the prediction errors for the pre improvement and post improvement BPNN training times were 0.023 and 0.016, respectively. When the training frequency of the BPNN before and after improvement was 550, the prediction errors were 0.0153 and 0.003, respectively.

The above data indicate that the improved BPNN has better performance in predicting heavy metal content in environmental soil, especially when the sample size is small or the training frequency is high. This provides a strong support for the improvement and optimization of environmental soil heavy metal control methods, which can improve accuracy and efficiency, and contribute to more effective management and control of heavy metal content in soil.

### Prediction accuracy of cadmium

Cadmium is a heavy metal. In the case of high concentration cadmium poisoning, it can cause serious damage to plant leaves, leading to growth retardation and plant dwarfism. This can hinder root development, leading to ecological barriers, decreased yield, and even death. Humans suffer from osteoporosis after eating these cadmium contaminated foods, and cadmium also leads to kidney damage, diabetes, lung damage, and even cancer, teratogenicity and mutation. Therefore, predicting the content of cadmium is very important and can be treated in a timely manner.

**Figure 2:**
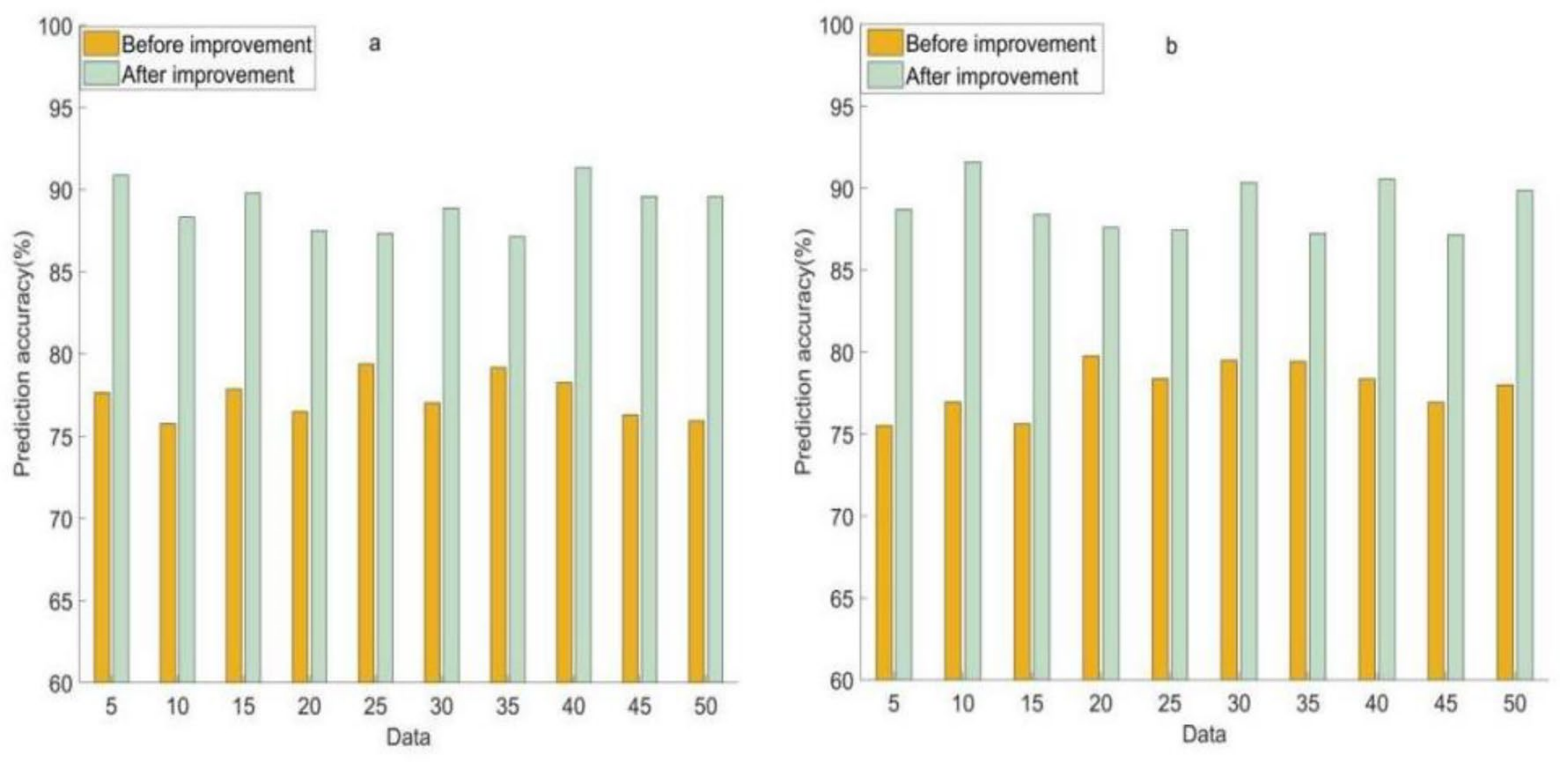
Prediction accuracy of soil cadmium content using BPNN before and after improvement in two experiments. (**a**) Prediction accuracy of cadmium content using BPNN before and after improvement in the first experiment. (**b**) Prediction accuracy of cadmium content using BPNN before and after improvement in the second experiment.

To ensure the rigor of the experiment, the pre and post improvement BPNN was subjected to two repeated experiments on the sample data. The accuracy of BPNN prediction of soil cadmium content before and after improvement in two experiments is shown in Fig. 2.

As shown in Fig. 2: Fig. 2a shows that in the first experiment, when the soil sample data was 5, the prediction accuracy of the BPNN for cadmium before and after improvement was 77.64% and 90.86%, respectively. When the soil sample data was 50, the prediction accuracy of the BPNN for cadmium before and after improvement was 75.95% and 89.56%. The lowest prediction accuracy of BPNN for cadmium was 75.77% before and 87.14% after improvement, respectively.

Figure 2b shows that in the second experiment, when the soil sample data was 5, the prediction accuracy of the BPNN for cadmium before and after improvement was 75.52% and 88.71%, respectively. When the soil sample data was 50, the prediction accuracy of the BPNN for cadmium before and after improvement was 77.99% and 89.85%. The lowest prediction accuracy of BPNN for cadmium was 75.52% before improvement and 87.15% after improvement, respectively.

The improved BPNN is a multi-level network structure form, where data and information are stored in different positions of the network and connected through numerous neurons. Therefore, in the event of local network faults or partial information distortion, the path and information can be automatically selected without affecting the operation of the entire network.

The above data reflect that the improved BPNN is significantly better than the improved BPNN in predicting the accuracy of cadmium content in soil. This indicates that the improved model may have more application potential in environmental soil heavy metal control methods, because it can provide higher accuracy under different sample data quantities, especially in the case of small sample data. This will help manage and control heavy metals such as cadmium in the soil more effectively.

### Prediction time for cadmium

In the improved BPNN, single neurons with simple structures are interconnected, and its data processing ability is unmatched by other algorithms, providing diverse and reliable data prediction methods for soil heavy metal content.

The average prediction time of cadmium using BPNN before and after improvement in the first experiment is illustrated in Table 2.

**Table 2:** Average prediction time of cadmium using BPNN before and after improvement in the first experiment (s).

Data	Before improvement	After improvement
5	30.97	21.66
10	31.08	20.36
15	29.36	20.64
20	32.16	20.99
25	28.25	23.73
30	27.68	20.05
35	25.45	20.14
40	30.90	21.96
45	32.44	21.72
50	29.04	23.81

As shown in Table 2: in the first experiment, the average prediction time of cadmium by BPNN before and after improvement was 30.97 s and 21.66 s in 5 soil sample data. Among 50 soil sample data, the average prediction time of cadmium by BPNN before and after improvement was 29.04 s and 23.81 s. In the first experiment, the average prediction time of the improved BPNN for cadmium was lower than the average prediction time of the BPNN for cadmium before the improvement.

The average prediction time of cadmium using BPNN before and after improvement in the second experiment is illustrated in Table 3.

**Table 3:** Average prediction time of cadmium using BPNN before and after improvement in the second experiment (s).

Data	Before improvement	After improvement
5	25.04	23.61
10	23.27	22.61
15	24.89	24.69
20	23.83	21.76
25	25.11	23.51
30	23.94	23.15
35	24.44	25.18
40	22.29	22.56
45	23.23	23.07
50	22.23	23.92

As shown in Table 3, in the second experiment, the average prediction time of cadmium by BPNN before and after improvement was 25.04 s and 23.61 s in 5 soil sample data. Among 50 soil sample data, the average prediction time of cadmium by BPNN before and after improvement was 22.23 s and 23.92 s.

The above data reflect that the improved BPNN shows a shorter average prediction time when predicting cadmium content in soil. Compared with the improved BPNN, the improved model has a significant improvement in efficiency. This is important for controlling heavy metals in environmental soil. Because being able to make predictions faster helps to quickly implement control measures to reduce the potential harm of heavy metals to the environment and ecosystems. The improved BPNN not only improves accuracy but also improves efficiency, which provides a better tool for soil heavy metal control.

### Prediction accuracy of lead

**Figure 3:**
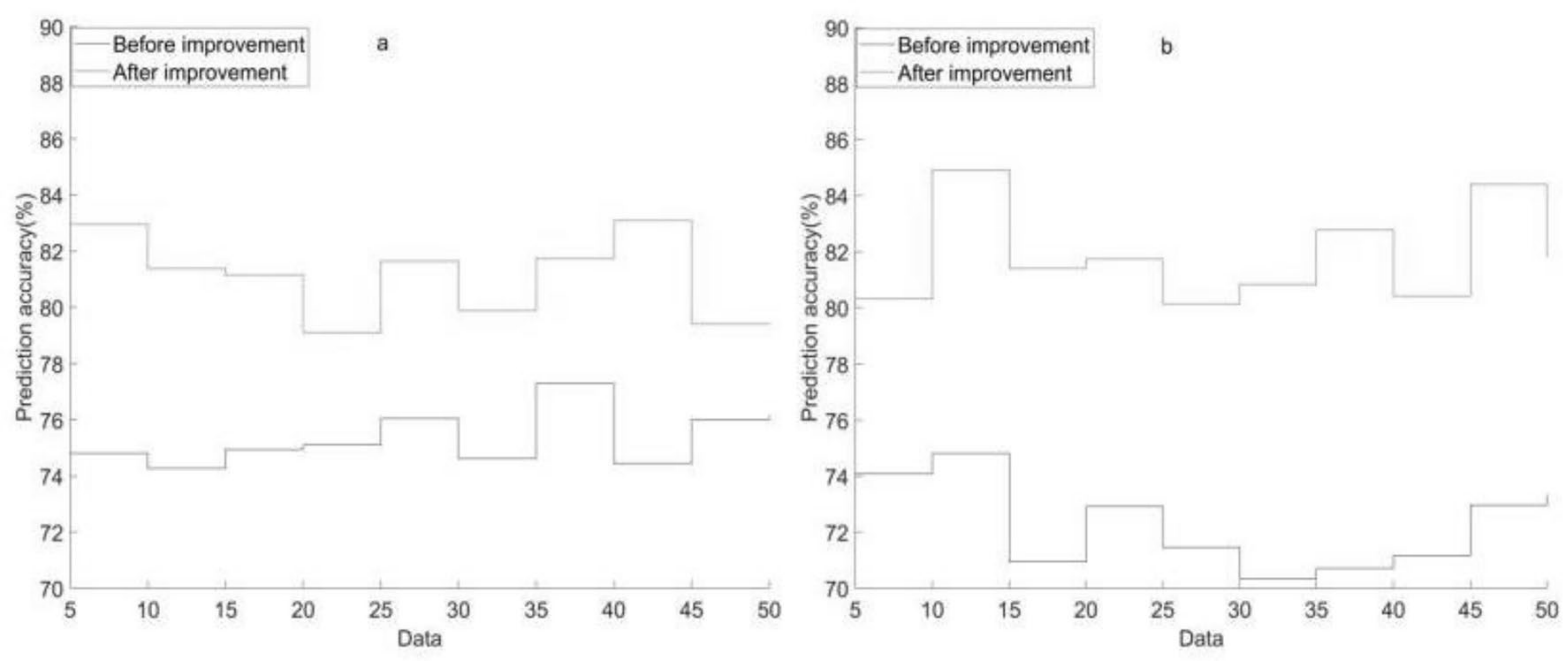
Prediction accuracy of BPNN for lead before and after improvement in two experiments. (**a**) Prediction accuracy of BPNN for lead before and after improvement in the first experiment. (**b**) Prediction accuracy of BPNN for lead before and after improvement in the second experiment.

The accuracy of BPNN prediction of soil lead content before and after improvement in two experiments is illustrated in Fig. 3.

As shown in Fig. 3: In the first experiment in Fig. 3a, when the soil sample data was 5, the prediction accuracy of the BPNN for lead before and after improvement was 74.82% and 82.95%, respectively. When the soil sample data was 50, the prediction accuracy of BPNN for lead before and after improvement was 76.15% and 79.44%. The lowest prediction accuracy of BPNN for lead before and after improvement was 74.27% and 79.09%, respectively.

Figure 3b shows that in the second experiment, when the soil sample data was 5, the prediction accuracy of BPNN for lead before and after improvement was 74.09% and 80.31%, respectively. When the soil sample data was 50, the prediction accuracy of BPNN for lead before and after improvement was 73.34% and 81.81%. The lowest prediction accuracy of BPNN for lead was 70.34% before and 80.13% after improvement, respectively.

The above data reflect that the improved BPNN is significantly better than the improved BPNN in predicting the accuracy of lead content in soil. This indicates that the improved model may have more application potential in environmental soil heavy metal control methods, because it can provide higher accuracy under different sample data quantities, especially in the case of small sample data. This will enable more effective management and control of heavy metals such as lead in the soil to safeguard the environment and human health. The improved BPNN not only improves accuracy but also improves efficiency, which provides a better tool for soil heavy metal control.

### Prediction time for lead

The pre improved BPNN used error gradient descent to determine data training errors, which has disadvantages such as slow convergence speed and easy falling into local extremum, especially when approaching the extremum, the training speed is slower. The improved BPNN incorporates momentum term, making data prediction more accurate and feasible.

**Figure 4:**
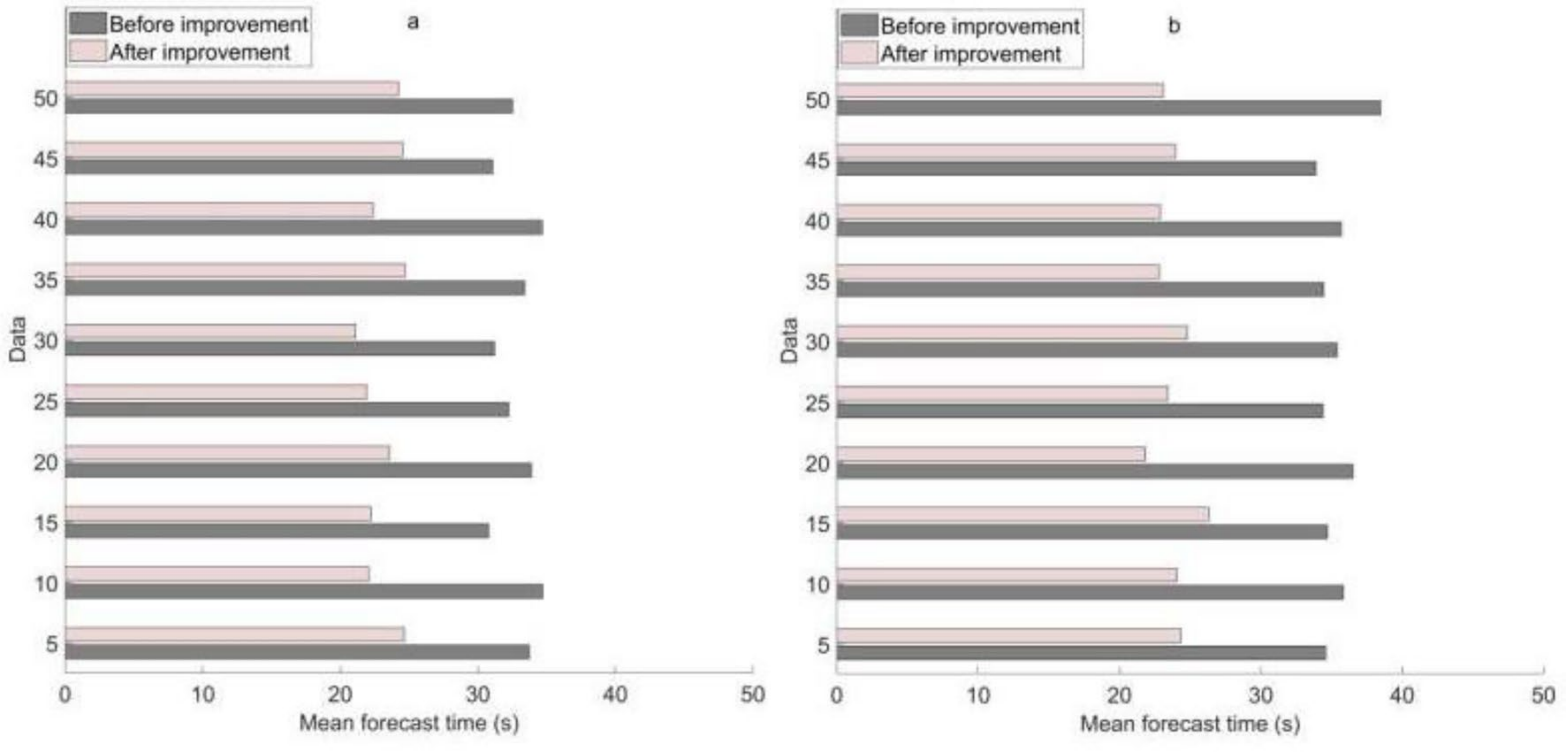
Average prediction time of lead using BPNN before and after improvement in two experiments. (**a**) Average prediction time of lead using BPNN before and after improvement in the first experiment. (**b**) Average prediction time of lead using BPNN before and after improvement in the second experiment.

To test the predictive performance of the BPNN before and after improvement, 50 sample data were predicted. The average prediction time of soil lead content using BPNN before and after improvement in two experiments is shown in Fig. 4.

As shown in Fig. 4: in the first experiment in Fig. 4a, the average prediction time of lead in five soil sample data using the pre improvement and post improvement BPNN was 33.73 s and 24.65 s, respectively. The average prediction time of lead in 50 soil sample data using BPNN before and after improvement was 32.51 s and 24.23 s, respectively.

In the second experiment in Fig. 4b, the average prediction time of lead in five soil sample data using the pre improved and post improved BPNN was 34.58 s and 24.33 s, respectively. The average prediction time of lead in 50 soil sample data using BPNN before and after improvement was 38.45 s and 23.09 s, respectively.

The above data reflect that the improved BPNN shows a shorter average prediction time when predicting lead content in soil. Compared with the improved BPNN, the improved model has a significant improvement in efficiency. This is important for environmental soil heavy metal control methods, as being able to make predictions faster helps to quickly implement control measures to reduce the potential harm of heavy metals to the environment and ecosystems.

## Treatment methods for heavy metals in soil

After comparing the prediction effects of the improved BPNN before and after the improvement, it was found that the improved BPNN had a better prediction effect on heavy metals. Therefore, after using the improved BPNN for heavy metal prediction, the following methods were selected based on the actual situation to treat soil heavy metals.

### Physical restoration method

Physical remediation method is a method of using liquids to remove pollutants from soil. The leaching process can be divided into two types: in-situ leaching method and ectopic leaching method. If the soil permeability is poor, it cannot automatically achieve the standard effect of in-situ leaching and filtration. The advantage of the ectopic impregnation method is that with the help of corresponding machines, the soil is loosened and mixed with the impregnation solution. After mixing, the treatment effect is very good, fully meeting the standards after treatment. Compared with the in-situ impregnation method, although the cost is higher, the treatment effect can meet engineering needs. The ex situ impregnation method can completely remove the heavy metal elements in the soil, but it has a certain impact on the tectonics and apparent form of the soil. This method not only increases the cost of excavation, transportation, preservation and disposal in the project operation, but also causes the loss of the matrix, which is only suitable for the soil with high permeability such as sandy soil.

### Chemical remediation

Chemical remediation utilizes chemical reagents to separate heavy metals from sediment through reactions such as oxidation, reduction, precipitation, and polymerization, or to transform them into non-toxic chemical forms. Traditional chemical remediation techniques have problems such as high consumption of chemicals and difficulty in operation, and some solidification agents can also cause certain pollution to the soil, thereby affecting the growth and development of other plants. The main contents of modern chemical remediation include chemical leaching technology, chemical immobilization technology, chemical oxidation–reduction technology, etc. Chemical redox has the characteristics of being less affected by the type and concentration of pollution. Chemical redox is aimed at heavy metals. It has a wide distribution range, a deep depth and is vulnerable to the impact of restitution.

### Microbial remediation

The utilization of specific microorganisms for adsorption, sedimentation, oxidation, reduction and other treatment of soil can effectively remove heavy metals in soil. On this basis, appropriate microbial remediation technology is studied and applied to various environmental pollution problems. The activity of microorganisms in soil mainly involves three processes: dissolution deposition, adsorption enrichment, and oxidation reduction. There are two basic principles in the remediation process of microorganisms in the environment: biological oxidation reduction and biological adsorption. Among them, biological oxidation–reduction refers to the process of causing heavy metals in soil to undergo oxidation–reduction reactions under the action of microorganisms, in order to alleviate or eliminate their pollution to the environment.

The principle of biosorption: in the laboratory, multi-stage amplification cultivation is carried out through experimental equipment such as a constant temperature incubator and a shaking table on the basis of experimental species in industry. The development prospect of microbial remediation technology is very broad. From the perspective of environmental treatment, compared with physical and chemical remediation methods, microbial remediation is less likely to cause pollution, and the treatment effect of pollutants is better. The self reproduction ability of microorganisms in the soil can also ensure that they can be treated for a long time, and achieve the expected effect, and finally achieve pollution control.

### Phytoremediation

In the plant treatment method, the absorption effect of heavy metals by plants is particularly significant. Some plants adsorb heavy metals by accumulating them in their roots, stems, and leaves, and then harvesting the plants to complete the treatment of heavy metals in the soil, achieving the expected treatment effect of heavy metals in the soil. Phytoremediation is characterized by low cost, good economic benefits, little interference to the soil environment, no change in the soil structure, no secondary pollution, and recovery of some heavy metals. Therefore, it has a wide range of applications, and also has indirect effects such as increasing soil permeability and reducing topsoil loss. However, this method also has certain drawbacks. Due to the scarcity of wild plant species, the super enriched plants screened in the field are generally relatively short and grow slowly. Affected by local climate, soil, and other factors, the restoration and control effect is poor and the cycle is long. Therefore, the promotion and use of this technology are greatly limited. Currently, research on excessive accumulation plants mainly focuses on the root system, and the repair time is relatively long. Therefore, it is very difficult to thoroughly purify the heavy metals in soil by using ultra concentrated phytoremediation, which may be unreasonable in terms of economic benefits.

### Plant microbial joint remediation

The combination of plants and microorganisms is currently the most promising remediation method. In the case of complex environment, bioremediation technology not only has to overcome its own shortcomings, but also produces some new problems and phenomena. It is necessary to screen and enrich remediation bacteria, establish a bacterial resource library, and strengthen research on microbial metabolic pathways. The plant-microorganism combined remediation technology can combine plants or microorganisms to achieve better governance effects.

Plants provide an attachment for microorganisms, which can improve the soil and make plant growth more prosperous. Nowadays, the combination of microorganisms and plants is more commonly used in engineering operations, which not only completes pollutant treatment but also beautifies the landscape. Environmental soil heavy metal control based on artificial neural network can make the following contributions:

- Heavy metal pollution monitoring: neural networks can be used to effectively monitor heavy metal pollution in soil. They can analyze vast amounts of soil properties and monitoring data to identify potential pollution hotspots and help detect problems early.
- Accurate prediction of heavy metal content: neural networks can provide accurate prediction of heavy metal content, helping to determine the concentration of different heavy metal elements in the soil. This is essential for developing effective governance measures.
- Sustainable soil management: neural networks can be used for long-term soil management decisions. They can help assess soil quality, monitor the effects of improvements, and develop sustainable soil conservation policies.

## Conclusions

Currently, there is an increasing global investment in soil heavy metal remediation. In order to find a simple and accurate method for soil heavy metal remediation, research on soil heavy metals is accelerating globally. This article explored the application of ANN in the treatment of heavy metals in environmental soil, and analyzed the predictive effect of BPNN on heavy metals in soil before and after improvement. After experiments, it was found that the improved BPNN had better prediction performance for both cadmium and lead than the previous BPNN. When the soil sample data was 50, the prediction accuracy of the BPNN for cadmium before and after improvement was 75.95% and 89.56%. The lowest prediction accuracy of BPNN for cadmium was 75.77% before and 87.14% after improvement, respectively. Validation of the BPNN’s predicted values is necessary in most cases, and validation can help evaluate the performance and accuracy of the BPNN model. By comparing the predicted values of the model with the actual observed values, it is possible to determine whether the model is able to provide acceptable predictions.

BPNN can process data in real time, quickly identify anomalies, and issue alerts, helping to take timely control measures to mitigate the impact of heavy metal pollution on the environment. This is essential for emergency response and emergency measures. After comparing the prediction effect of the improved BPNN on heavy metals in soil, different treatment methods can be selected according to the actual situation of soil, such as physical hair, chemical repair, microbial repair, phytoremediation, and plant microbial joint repair. The analysis of this article has provided important reference value for the research direction of soil heavy metal management, and it is expected to provide a more effective and sensitive method in the future. Meanwhile, for certain regions, due to complex terrain conditions and difficult sampling, an improved BPNN can be used for prediction, thereby reducing sampling and analysis costs, and reducing chemical pollution in the analyzed samples.

## Data Availability

The datasets used and/or analysed during the current study available from the corresponding author on reasonable request.
